# Loss of the Kidney Urate Transporter, Urat1, Leads to Disrupted Redox Homeostasis in Mice

**DOI:** 10.3390/antiox12030780

**Published:** 2023-03-22

**Authors:** Neema Jamshidi, Kabir B. Nigam, Sanjay K. Nigam

**Affiliations:** 1 Department of Radiological Sciences, University of California, Los Angeles, CA 90095, USA; 2 Institute of Engineering in Medicine, University of California, San Diego, La Jolla, CA 92093, USA; snigam@ucsd.edu; 3 Department of Psychiatry, Brigham and Women’s Hospital, Boston, MA 02130, USA; 4 Department of Psychiatry, Harvard Medical School, Boston, MA 02130, USA; 5 Departments of Pediatrics and Medicine (Nephrology), University of California, San Diego, La Jolla, CA 92093, USA

**Keywords:** metabolic network reconstruction, genome-scale, constraint-based optimization, flux simulation, remote sensing and signaling theory, ABCG2, SLC2A9, SLC22 transporter

## Abstract

High uric acid is associated with gout, hypertension, metabolic syndrome, cardiovascular disease, and kidney disease. URAT1 (SLC22A12), originally discovered in mice as Rst, is generally considered a very selective uric acid transporter compared to other closely-related kidney uric acid transporters such as OAT1 (SLC22A6, NKT) and OAT3 (SLC22A8). While the role of URAT1 in regulating human uric acid is well-established, in recent studies the gene has been linked to redox regulation in flies as well as progression of renal cell carcinoma. We have now identified over twenty metabolites in the Urat1 knockout that are generally distinct from metabolites accumulating in the Oat1 and Oat3 knockout mice, with distinct molecular properties as revealed by chemoinformatics and machine learning analysis. These metabolites are involved in seemingly disparate aspects of cellular metabolism, including pyrimidine, fatty acid, and amino acid metabolism. However, through integrative systems metabolic analysis of the transcriptomic and metabolomic data using a human metabolic reconstruction to build metabolic genome-scale models (GEMs), the cellular response to loss of Urat1/Rst revealed compensatory processes related to reactive oxygen species handling and maintaining redox state balances via Vitamin C metabolism and cofactor charging reactions. These observations are consistent with the increasingly appreciated role of the antioxidant properties of uric acid. Collectively, the results highlight the role of Urat1/Rst as a transporter strongly tied to maintaining redox homeostasis, with implications for metabolic side effects from drugs that block its function.

## 1. Introduction

Disorders of uric acid are widespread and are associated with gout, kidney stones, metabolic syndrome, hypertension, kidney disease, and cardiovascular disease [1]. Under normal circumstances, much of uric acid handling (tubular secretion and reabsorption) occurs in the proximal tubule of the kidney and, to a lesser degree, in the intestine and other tissues. Numerous lines of evidence-from in vitro studies, GWAS, and mouse knockouts-indicate that over a dozen SLC and ABC transporters in the kidney and intestine play a key role in urate homeostasis. Furthermore, in the setting of chronic kidney disease (CKD), intestinal extrusion of uric acid becomes relatively more important-presumably through inter-organ crosstalk with the injured kidney (resulting in increased expression and/or activity of ABCG2) [2,3]. This is considered a human example of small molecule remote inter-organ communication via urate transporters, as proposed in the Remote Sensing and Signaling Theory (RSST) [4,5,6,7]. In the normally functioning kidney, uric acid handling appears to depend upon both multi-specific “drug” transporters (e.g., OAT1 or SLC22A6, OAT3 or SLC22A8) and transporters with high selectivity for uric acid (e.g., URAT1 or SLC22A12, SLC2A9). Like OAT1 and OAT3, URAT1 is a SLC22 transporter [8]; indeed, several other SLC22 family members, including OAT4 (SLC22A10) and OAT10 (SLC22A13), are also uric acid transporters. However, URAT1, originally described in mice as RST, has received a great deal of attention because mutations are associated with exercise-induced kidney stones and many single nucleotide polymorphisms have been associated with hyperuricemia [9,10].

However, while deletion of Urat1/Rst in knockout (KO) mice resulted in the expected effect on renal urate handling, this effect was modest [11]. Because of the known roles of the closely-related OAT1 and OAT3 in regulating or modulating many aspects of metabolism [12,13,14], untargeted metabolomics was performed on the Urat1KO plasma when the KO data was first published in 2008. This suggested several small molecule metabolites to be elevated, but they could not be clearly identified at the time. Nevertheless, this raised the possibility that Urat1 might regulate additional metabolic processes. For example, whereas URAT1 is well known to regulate uric acid levels in humans, the gene has been associated with an altered response to oxidative stress in flies [15], the progression of renal cell carcinoma [16,17], and even in COVID-19 outcomes [18]. This suggests that URAT1 loss results in a systemic effect on overall cell/tissue function, potentially affecting redox regulation and cell growth.

Here, we have performed a more targeted analysis of the Urat1 knockout, which has revealed the presence of 8 new metabolites. Interestingly, this set of metabolites is different from metabolites that are elevated in the Oat1 and Oat3 knockout mice; chemoinformatics and machine learning analyses revealed that the metabolites also differ in their general structures and molecular properties. To understand the overall metabolic impact of these alterations, we first performed standard metabolite-based pathway analysis. Analysis of the resulting models revealed seemingly disparate changes in metabolism from loss of Urat1. At the single pathway level, loss of Urat1 was implicated in metabolism of fatty acids, amino acids, pyrimidines, and sugars. Nevertheless, deeper analysis using multi-omics constraint-based modeling with Constraint-based Reconstruction Analysis (COBRA) of genome-scale models (GEMs) revealed that the parts of the different sub-systems that were affected were related to maintenance of the redox state. Moreover, increases in catalase and peroxidation reactions were noted with Urat1/Rst loss. In light of the widely held view of uric acid as an antioxidant, it is, to our knowledge for the first time, possible to appreciate the implication of the redox state changes revealed by multiscale metabolic reconstructions. These results set the stage for new studies on how endogenous biochemical pathways modulated by Urat1, including fatty acid and sugar metabolic pathways involved in metabolic syndrome, regulate redox state at the cell, tissue and whole organism level. Furthermore, since inhibitors of Urat1 are increasingly used to treat human hyperuricemia, the clarification of the role of Urat1 in individual in vivo metabolic pathways as well as overall redox state should be helpful understanding potential drug-metabolite interactions and unexpected toxicity from URAT1 inhibiting drugs.

## 2. Materials and Methods

### 2.1. Wildtype and RST Knockout Mice

All experimental protocols involving the use of animals were approved by the UC San Diego Institutional Animal Care and Use Committee (IACUC). All animals were handled in accordance with the Institutional Guidelines on the Use of Live Animals for Research. Adult WT and Urat1 (Rst) knockout males [19] were housed separately under a 12-h light–dark cycle; the animals were provided *ad libitum* access to food and water. Serum was obtained from the whole blood. Mice were adults at least 2 months of age. These animals have been described in previous publications [11].

### 2.2. Transcriptomic Profiling

Microarray profiling and analyses were performed as previously described [11]. Briefly, RNA prepared from wild-type and knockout kidneys was purified on RNeasy columns (Qiagen, Valencia, CA, USA) followed by either to linear amplification microarray analysis. The amplified RNA was labeled by incorporation of biotinylated nucleotides during in vitro transcription and then hybridized to Affymetrix microarrays, washed, and scanned per the standard Affymetrix protocol.

### 2.3. Metabolomic Profiling

Individual, unpooled samples were measured by the Metabolon analytical system (Metabolon, Inc., Durham, NC, USA). The samples were subjected to ultrahigh performance liquid chromatography-tandem mass spectroscopy (UPLC-MS/MS) utilizing an ACQUITY ultra-performance liquid chromatography (UPLC) (Waters, Milford, MA, USA) and a Q-Exactive high resolution/accurate mass spectrometer interfaced with a heated electrospray ionization (HESI-II) source and Orbitrap mass analyzer operated at 35,000 mass resolution (Thermo Scientific, Waltham, MA, USA). To calculate the p-values, two-way analysis of variance (ANOVA) testing was used [20].

### 2.4. Chemoinformatics and Machine Learning

The methods for analyzing small molecule molecular properties using ICM Chemist (Molsoft) and for narrowing down to a set of relevant molecular properties have been previously described in considerable detail [21,22]. Six metabolites were significantly elevated at *p* < 0.05 in the Urat1/Rst knockout plasma (Supplementary Materials Table S1). This was deemed an insufficient number to perform chemoinformatics analysis, so we included metabolites that were elevated with *p* < 0.1 for which the relevant molecular properties could be obtained. This resulted in a total of 20 metabolites. These were compared to molecular properties from a randomly selected sample of 20 metabolites elevated in the Oat1 knockout mouse and the Oat3 knockout mouse. The Freeviz analysis was performed in Orange [23].

### 2.5. Modeling and Analysis

Multiple genome-scale metabolic reconstructions were used to construct a scaffold for data mapping and simulation including Recon3D and multiple microbial reconstructions. Recon3D is the most recent iteration of the human metabolic network reconstruction and has more than twice the number of genes as Recon1 and more than three times the number of biochemical reactions/transporters than Recon1 [24,25]. The Gene Inactivity Moderated by Metabolism and Expression (GIMME) algorithm maps gene expression data as reaction weightings and calculates context-specific subnetworks to achieve particular metabolic objectives using FBA [26]. These different networks are then compared to calculate a ‘consistency score’ that is minimized to optimize alignment of the resultant network with the gene expression data. General description: $S\in R^{mxn},\left\{v,c\right\}\in R^{n}$,

(1) $$min(c^{T}-|v|)$$

subject to:

(2) S·v=0

(3) $$v^{l}\leq v\leq v^{u}$$

with $c=x_{cutoff}-x$ for c>0, else c=0. Conversion of the above to a linear programming problem is performed by constructing a convex null space by redefining the flux vector such that,

(4) $$v=v^{+}-v^{-}$$

(5) $$0\leq v^{+}\leq v^{u}$$

(6) $$0\leq v^{-}\leq -v^{l}$$

described in [26] and implemented in [27]. The mouse transcriptomic data were mapped to human orthologs using the NCBI HomoloGene Database (accessed on 27 October 2019). Since transcript expression levels and fluxes are qualitatively associated, but generally not quantitatively correlated, the GIMME algorithm was used to apply context-specification with the transcriptomic data for WT and KO model construction. As described previously, transcriptomic data were incorporated based on present/absent (P/A) calls using Affymetrix Microarray Suite Version 5.0 [12]. A minimum of 3 microarray datasets were included for each condition (WT and KO) and were analyzed separately; for a gene to be considered present, it had to be present in at least 2 of 3 sets of data. Since we were ultimately interested in the response to the Urat1 (Rst) KO and condition-specific measurements, substrate uptake was refined to only account for metaboliteswith redox cofactors dominating the connectivity analysi that were mapped from Recon3D to the experimental data. The metabolites were selected based on *p* < 0.05 for the principle analysis (we also relaxed the condition to *p* < 0.1 for consistency with the structural ML analysis and to ensure that the observations for the more stringent cutoff persisted). Metabolomic data constraints test with flux variability analysis (FVA) [28] to classify metabolites into three groups: those that can only be secreted, those that can only be taken up, and those that can be secreted or taken up. Interpretation of the metabolomic fold change measurements (KO relative to WT) were as follows:

▸ If KO/WT > 1 and the metabolite could only be secreted, then the metabolite exchange was constrained with a non-zero lower bound (10% of maximum secretion)
▸ If KO/WT > 1 and the metabolite could only be taken up, then the metabolite exchange was constrained with a lower bound that was greater than the minimum
▸ If KO/WT < 1 and the metabolite could only be secreted, then the metabolite exchange was constrained with an upper bound that was less than the maximum
▸ If KO/WT < 1 and the metabolite could only be taken up, then the metabolite exchange was constrained with upper bound that was less than zero (90% of maximum uptake)

Since the comparison focuses on alterations, an arbitrary reference uptake of 0.25 mM/gDW/h was specified for the Urat1 (Rst) KO and WT models. Additionally unconstrained uptake/secretion was permitted for oxygen, sodium, potassium, iron, magnesium, bicarbonate, protons, and water. Prior studies analyzing murine kidney transporter KO models comparing model content with different objective functions including biomass, urea, and ATP production resulted in models with similar composition and size. ATP production was used, given that the transcriptomic data analysis was performed on the organ level (as opposed to single-cell) transcriptomics. Following construction of two GIMME models (WT and KO), the feasible solution space of each model was sampled. Differentially active reactions between WT and KO were than computed from the normalized sampled feasible flux states [29], requiring *p* < 0.001 for the Kolmogorov-Smirnov test. The gpSampler function in the CobraToolbox was used to characterize the steady state solution space with 2n sampled points (for *n* model reactions as described above). Reaction co-sets were calculated from these points with a 0.95 Pearson correlation coefficient cutoff. Given the fact that reaction order can affect the composition of a minority of reactions in GIMME, we employed a strategy similar to the Model Building Algorithm (MBA [30]), in which we randomly re-ordered the reaction order and generated new models (10× for WT and KO) and only reported reactions that were shared among all 10 models (for WT and KO, respectively).

Model specific connectivity analyses were performed by modifying the stoichiometric matrix, S, as defined above. First S was converted to a binary matrix, $S_{b}\in {\left\{0,1\right\}}^{mxn}$, with all non-zero entries replaced by 1. Next all columns were zeroed out for the exception of the reactions of interest (e.g., the reactions present in KO but not WT, etc). Finally, the metabolite connectivity, $c_{m}$, was calculated as $c_{m}=diag\left(S_{b}\cdot S_{b}^{T}\right)$ (Supplementary Materials Tables S3–S6). Multiple methods for context specification have been described in the literature; we have found GIMME to provide the most robust results for complex mammalian models, however we also considered other methods for generating models, and found similar results [31] (Supplementary Materials Table S7). Model modifications and simulations were carried out with using CobraPy [32] and the CobraToolbox v2.0 [27] with the Gurobi Optimizer (v8.0). Network pathway maps were created using Escher Maps [33].

## 3. Results

The Urat1 (Slc22a12, Rst) KO mouse has previously been described [11]. It has a normal lifespan compared to wildtype (WT), and normal weight, normal $O_{2}$ consumption, normal $CO_{2}$ production, normal food consumption, normal water consumption, normal movement, normal blood pressure, normal hematocrit, and normal plasma sodium and potassium. Consistent with human data, the KO mouse has altered renal handling of uric acid [11,34]. In these earlier studies, untargeted plasma metabolomics revealed dozens of differences in features detectable by mass spectrometry; while a number of m/z ratios were in the 250–350 range, the metabolites could not be identified within the databases of the time (i.e., circa 2008). Thus, even then, there was the suspicion that Urat1 might be involved in metabolic pathways not directly related to urate transport, *per se*.

We have now analyzed the plasma metabolome of the Urat1 KO using a targeted approach measuring over 500 identifiable molecules. This provides a portrait of the systemic physiological changes due to the loss of that portion of urate reabsorption dependent upon Urat1 function. To create a more comprehensive portrait of local as well as systemic changes from the loss of Urat1, we first analyzed the metabolite alterations in KO mouse serum. We then sought to determine the molecular properties of the metabolites accumulating in the Urat1 (Rst) knockout mice and used machine learning methods to determine whether particular sets of molecular properties could help distinguish metabolites altered in the Urat1KO compared with those altered in the Oat1KO and Oat3KO. Finally, to determine functional metabolic consequences of Urat1 KO, we constructed context-specific GEMs using transcriptomic and metabolomic data, comparing the metabolic flux states in WT versus KO mice. Such reconstructions of metabolic networks based in multi-omics data can define, in the context of in vivo gene deletion (i.e., Urat1), alterations in metabolic capacities at multiple scales that are not evident from conventional pathway analysis. Indeed, it can, as is the case here, reveal how seemingly unrelated pathways cooperate, or function in a complementary manner, in the context of the whole tissue or organism.

### 3.1. Metabolite Alterations Resulting from Urat1 Deletion

**Figure 1:**
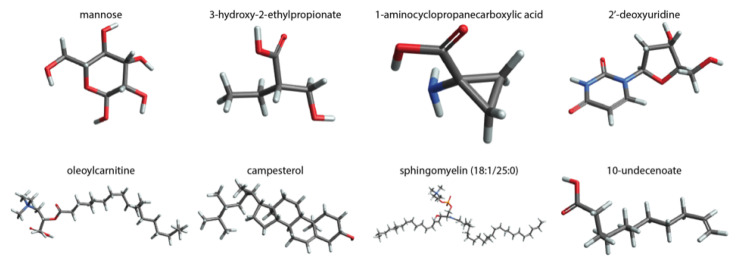
Names and structures of the eight metabolites with significantly altered fold changes (*p* < 0.05). Metabolites exhibited increased fold changes for KO versus WT ratios with the exception of sphingomyelin and campesterol (which were both decreased). Standard color schemes for the wire structures are used (e.g., carbon: gray, oxygen: red, nitrogen: blue, hydrogen: white).

Metabolomics revealed that 8 metabolites were altered (6 were elevated and 2 were decreased) in the Urat1KO plasma for *p* < 0.05 (and 22 metabolites for *p* < 0.1) (Figure 1 and Supplementary Materials Table S1). These included fatty acids (e.g., 10-undecanoate), fatty acyl carnitines (e.g., oleylcarnitine), amino acid derivatives (e.g., hydroxyasparagine), tryptophan metabolites likely derived from the gut-microbiome (kynurenine), pyrimidines (5-deoxyuridine), bile acids (tauroursodeoxycholate), and mannose. It is worth noting here that, because Urat1 is an apically (urine side) facing transporter involved in reabsorption urate, plasma metabolomics of the Urat1KO is less likely to reveal transported substrates compared plasma metabolomics of the knockouts of the basolateral (blood-facing) Oat1 and Oat3. For example, even for demonstrating altered urate handling in the original description of the Urat1KO, there were no significant changes in urate levels in the plasma. However, alterations in urate handling were evident when fractional excretion of urate was calculated [11]. In this study, there was also no significant change in urate in the Urat1KO. These experiments were designed to assess the tissue and systemic metabolic alterations due to loss of Urat1, and thus urine was not analyzed.

### 3.2. Chemoinformatics and Machine Learning Analysis of Metabolites Dependent Upon Urat1 Function *In Vivo*

**Figure 2:**
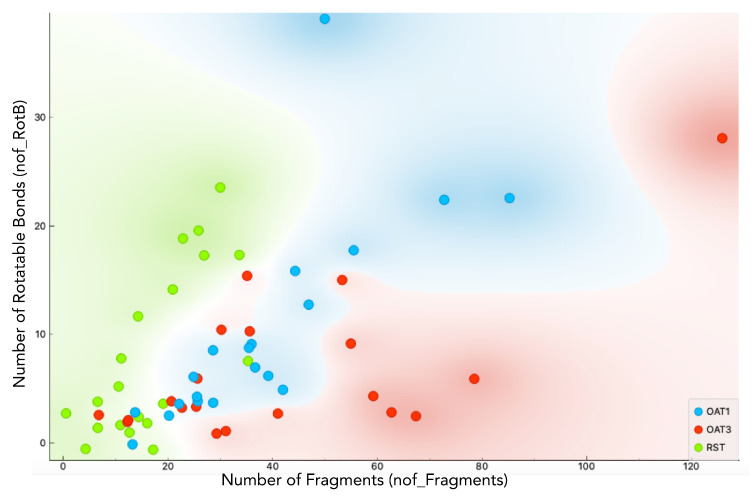
The scatter plot indicates how two molecular properties, the number of fragments (abscissa) and the number of rotatable bonds (ordinate), provide a reasonable separation of metabolites elevated in the 3 knockouts. Green circles represent the RST KO and the red and blue circles correspond to the Oat1 and Oat3 KO, respectively.

**Figure 3:**
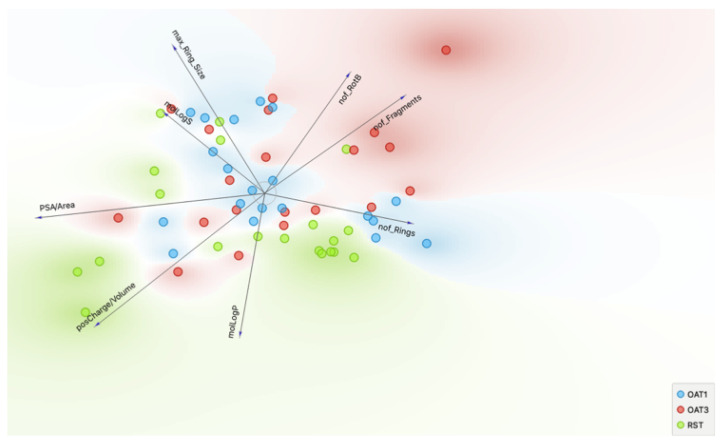
FreeViz vector plot of the molecular features of increased metabolites in OAT1, OAT3, and RST (Urat1). Each axis corresponds to a different physical/biophysical measurement; the length of each axis corresponds to the magnitude of the feature and the angle between any two axes corresponds to the correlation between the features [23]. Green circles represent the RST KO and the red and blue circles correspond to the Oat1 and Oat3 KO, respectively.

The “molecular properties” of Urat1KO metabolites were analyzed using ICM Chemist Pro (Molsoft) and then compared to molecular properties of Oat1KO and Oat3KO metabolites (Figure 2 and Figure 3). The metabolites chosen for this analysis were those that were uniquely present in the three KOs, reducing the total dataset to 174 metabolites. The reasons for approaching the machine learning analysis this way have been described previously [4]. While Oat3KO metabolites tended to have more rings and Oat3KO metabolites were more fragmentable, Urat1KO metabolites tended to have a more positive charge density (Figure 2 and Figure 3). Nevertheless, the differences in individual molecular properties described above for metabolites accumulating in the Urat1KO compared to the Oat1KO and Oat3KO need to be viewed as generalizations with many exceptions.

Instead of individual molecular properties, when we examined a set of molecular properties, the differences between the metabolites altered in the Urat1KO and the two Oat knockouts became even more evident. The chosen set of 8 molecular properties (molLogP, PSA/area, molLogS, nof_Rings, max_Ring_Size, nof_Fragments, posCharge/Volume, nof_RotB) was narrowed down from a much larger list of molecular properties by analyzing information gain using the Rank widget in the Orange data mining and machine learning software as well as various distributions of single molecular properties. Because the dataset was unbalanced (with only 20 Urat1KO metabolites compared to approximately 4 times as many in the Oat1KO and Oat3KO), for machine learning we compared all 20 Urat1KO metabolites to 20 randomly sampled from the Oat1KO and 20 randomly sampled from the Oat3KO). FreeViz visualization (Figure 3) of the differences between the Urat1 (Rst) KO metabolites and the two OatKOs based on the set of 8 molecular properties-with the length of each vector indicative of the importance of each molecular property and the angle between the vectors indicative of the degree of correlation between molecular properties, allowing for comparison and grouping of metabolites across the set of molecular properties. Even though there were no overlapping metabolites between the 60 metabolites represented (20 Urat1/Rst KO, 20 randomly sampled Oat1KO, 20 randomly sampled Oat3KO), there is some crossover between what is generally a Urat1 space defined by the 8 vectors (light green shaded space) and the Oat space (light red and light blue shaded space).

### 3.3. Metabolic Network Structural and Functional Differences of Wildtype and Urat1 Knockout Mice

In order to simultaneously analyze the metabolomic and transcriptomic alterations in a biologically integrated context and to discern how these disparate alterations in metabolic pathways fit together at the level of the whole tissue and organism, as well as to overcome the fact that a relatively small number of metabolites were altered in the Urat1KO, we used a well-validated systems approach for analysis of metabolism [35]. Genome-scale metabolic network reconstructions provide a means to carry out multi-omics, data-driven model construction; this enables flux-based simulation studies that evaluate the metabolic capabilities of the cells, tissues, and organisms [36,37]. GEMs are derived from highly-curated databases that encode gene-protein-reactions–in other words, the biological relationships among genes, the enzymes and transporters they encode, and the small metabolites that are transformed and transported [38,39]. Experimental data such as transcriptomics and metabolomics can be used to constrain enzyme and transport fluxes; this makes it possible to calculate the different flux states using omics data from the WT and KO [12,13,26,30,34,40,41,42,43]. This approach has been successful in delineating the effects of Oat1 and Oat3 KOs at multiple scales [12,13,43,44]; thus it could provide insights into the metabolomic changes seen in the Urat1KO.

**Figure 4:**
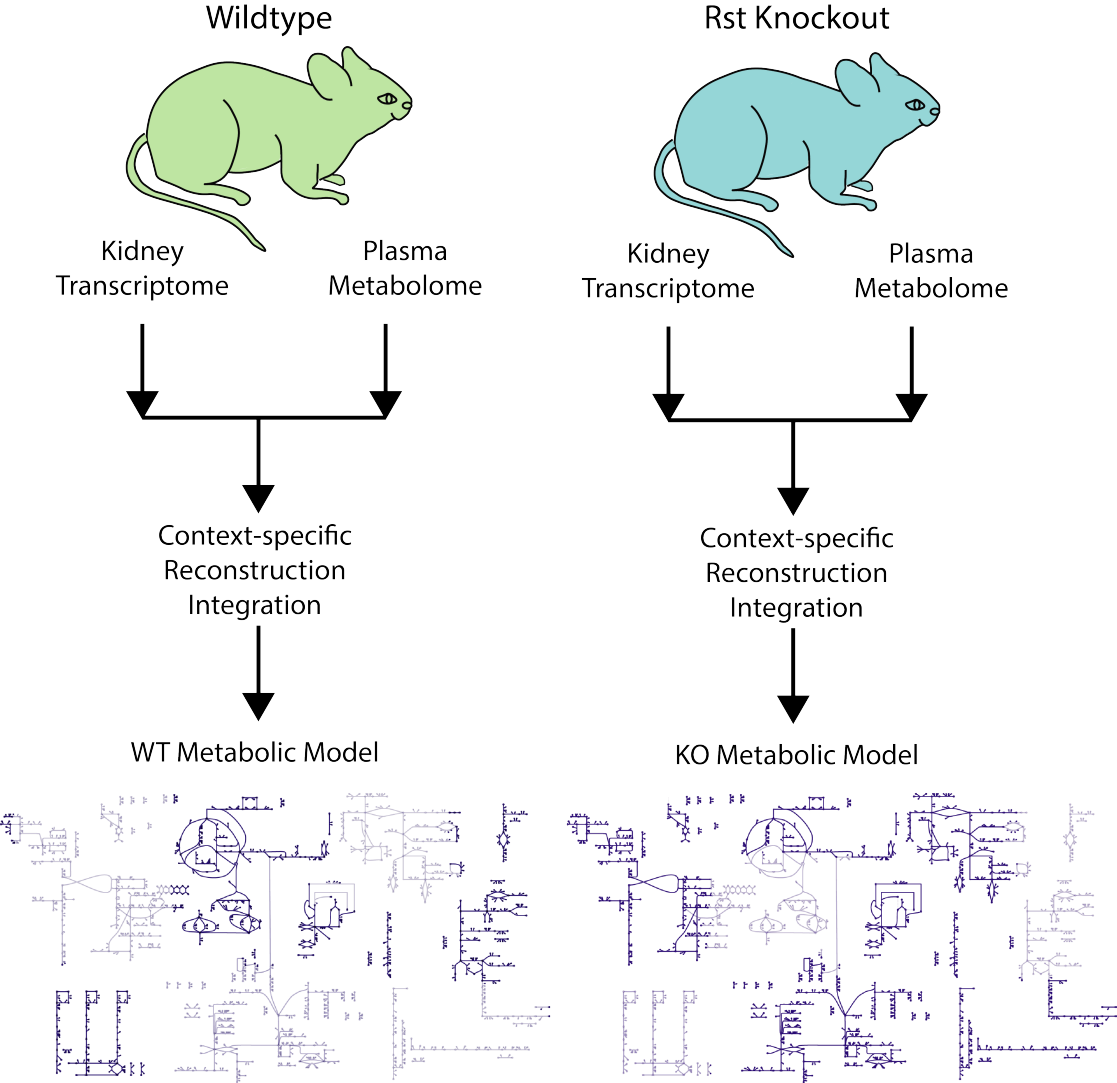
Renal transcriptomic data in conjunction with plasma metabolomic profiles of WT and Urat1/Rst KO mice were used to build in silico models of metabolism. Comparisons between the WT and KO states were then performed through assessment of differences in the content of the models (i.e., differences in the composition of the WT versus KO metabolic networks) as well as their functional states (i.e., comparison of the different attainable flux states through simulation and comparison of the feasible steady state flux distributions).

**Figure 5:**
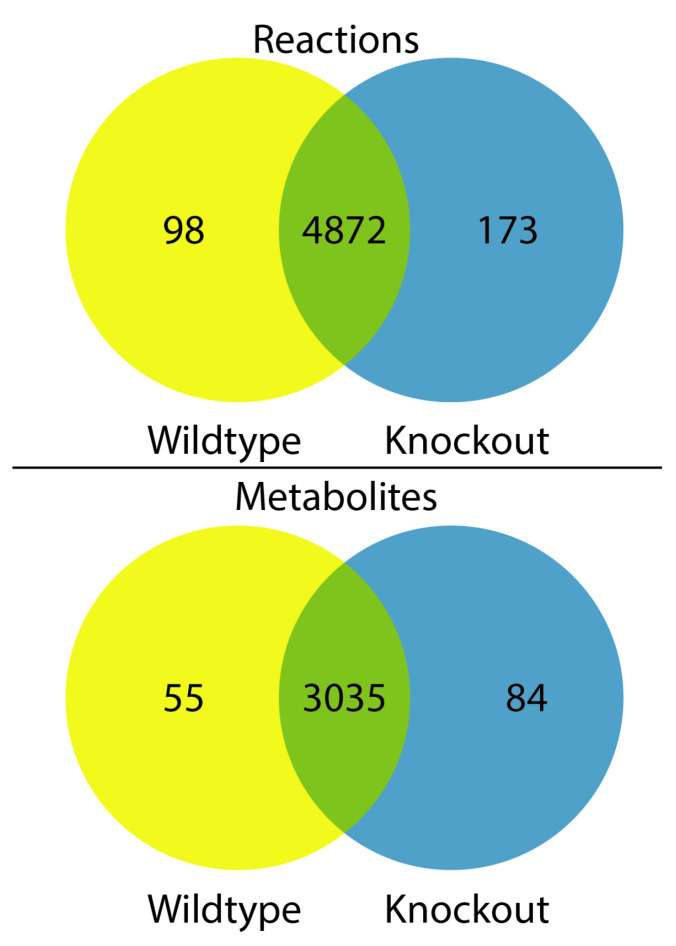
High level comparison between WT and Urat1/Rst KO GEMs. metabolic network content comparison highlighting the shared (**middle**, green), unique wildtype (**left**, yellow), and unique knockout (**right**, blue) reactions (**top**) and metabolites (**bottom**). The large majority of reactions and metabolites are shared between the two models and there are approximately twice as many reactions that are unique to the KO (‘gain of function’) in comparison to WT.

**Figure 6:**
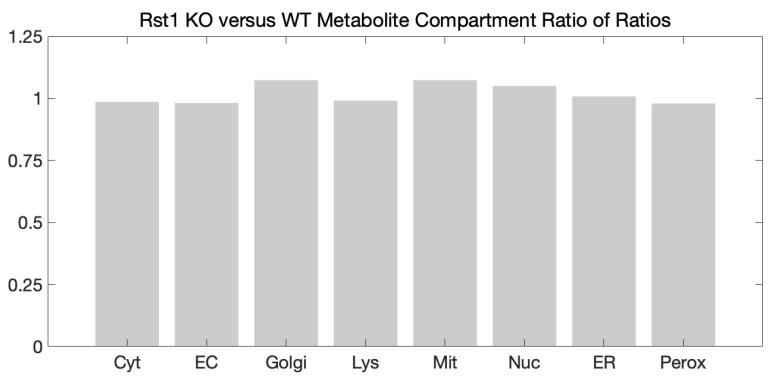
Comparison between WT and Urat1/Rst KO GEMs based upon intracellular compartment size. Ratio between the number of metabolites within each compartment of the Urat1KO versus WT GEMs. There are not significant differences among the metabolomic cohorts in in each model, consistent with the differences in metabolite composition of the two network models. Abbreviations are as follows: Cyt-cytoplasm, EC-extracellular, Golgi-Golgi apparatus, Lys-lysosome, Mit-mitochondria, Nuc-nucleus, ER-endoplasmic reticulum, Perox-peroxisome.

**Figure 7:**
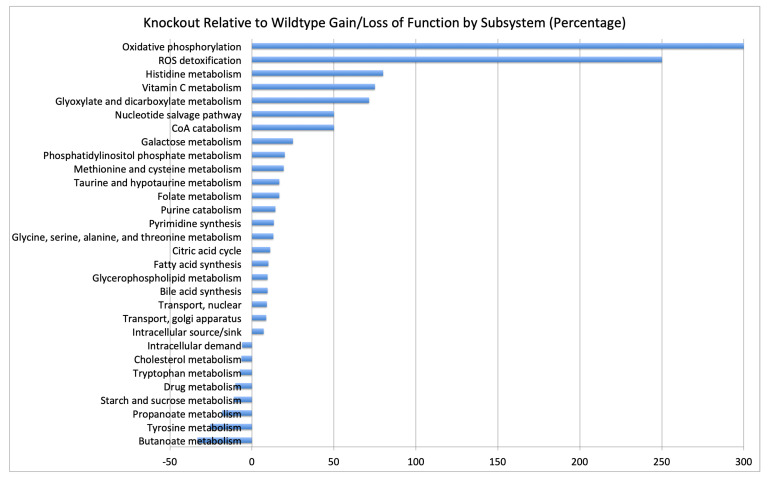
Comparison between Urat1/Rst KO and WT GEMs based on reaction differences according to subsystem classification. Reactive oxygen species handling and oxidative phosphorylation-related reactions exhibited the biggest changes. Further evaluation of the reactions implicating other metabolic sub-systems revealed that the changes were generally involved in redox reactions and cofactor charging.

The plasma metabolomics data from the Urat1KO can be viewed as a systemic analysis of altered metabolism in the Urat1KO. Nevertheless, the transporter mediating reabsorption of uric acid and possibly other organic anions is almost exclusively in the apical membrane of the proximal tubule of the kidney; thus, loss of the transporter is likely to affect intracellular levels of metabolites and thereby result in altered gene expression in kidney tissue. The metabolic reconstruction provides a portrait of altered local metabolism in these normally Urat1-expressing cells/tissue after deletion of the gene. This view, then, would complement the conventional analyses of altered individual pathways obtained by analyzing the plasma metabolites changed in the Urat1KO. Thus, in order to assess the transcriptomic and metabolomic changes in the Urat1KO on cellular flux states, we used constraint-based analysis approach with GEMs using Recon3D (Figure 4). The paired in silico transcriptomic and plasma metabolomic datasets resulted in the construction of a WT and Urat1KO metabolic network model (Figure 4). These two in silico models were then compared in terms of (1) reactions that were shared between the two models with significantly different reaction fluxes and (2) differences in reaction content between the two models (Figure 5). The size of the in silico WT and Urat1KO models (Figure 5) were noticeably smaller than genome-scale models generated for Oat1 and Oat3 models using similar datasets and methods [44]. Metabolomic differences within the different intra-cellular compartments were also assessed, but there were not significant variations in WT versus Urat1KO (Figure 6). Judging from the metabolomics data alone, this was not surprising, as the Oat1KO and Oat3KO exhibit broad changes across the metabolome, in which hundreds of significantly changed metabolites were detected [20,21]. An assessment of the altered subsystems based on reaction content differences between WT and Urat1KO GEMs (Figure 7) implied broad changes (30 altered metabolic subsystems). From the figure, it is immediately apparent that a large percentage of the Oxidative Phosphorylation and Reaction Oxygen Species (ROS) subsystems were involved. Since the size of the different subsystems could potentially bias the observed changes and artificially increase or decrease the perceived alterations due to Urat1KO, the individual reactions contributing to these changes were assessed.

### 3.4. Altered Tissue and Systemic Redox State in the Urat1KO Mouse

**Figure 8:**
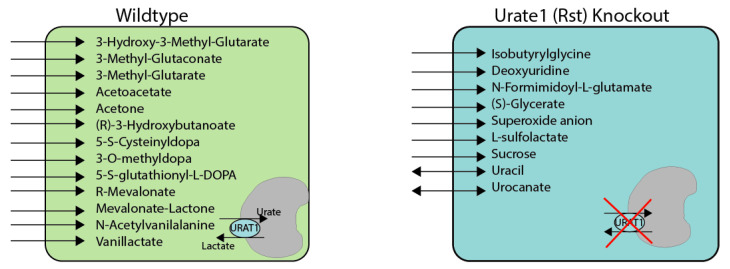
Summary comparison of the unique exchange metabolites for the WT (green, **left**) and Urat1KO (blue, **right**) models. Loss of URAT1 functionality appears to be accompanied by increased uptake of multiple metabolites that are directly or indirectly linked to redox cofactor balances. Although URAT1 in the kidney proximal tubule is apical, the transport mechanisms correspond to the predicted uptake of metabolites may be apical and/or basolateral and may occur in different organs.

**Figure 9:**
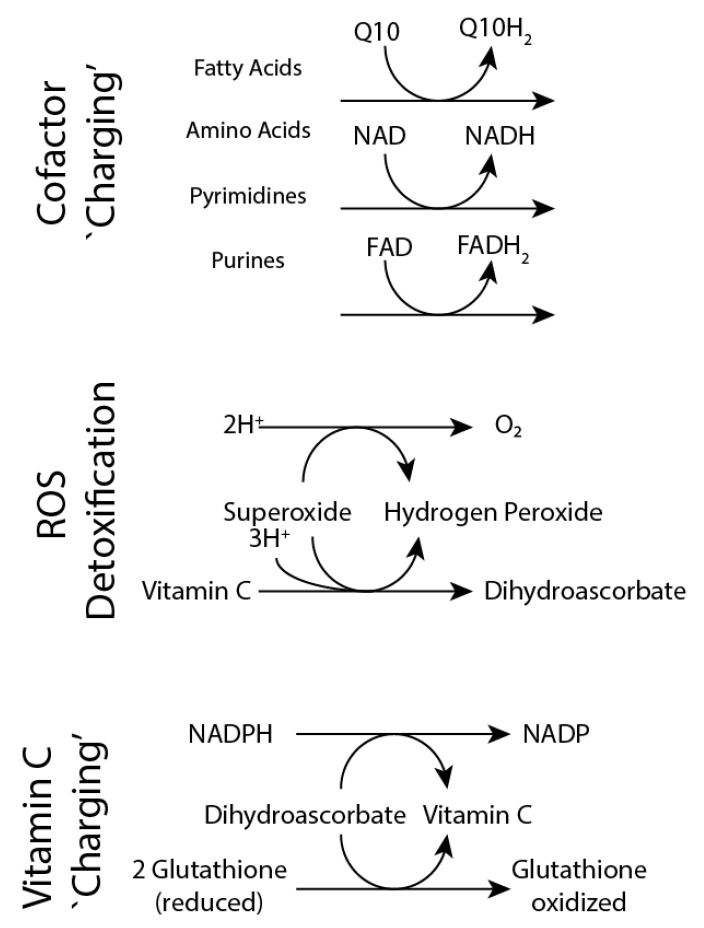
Detailed evaluation of the unique sets of reactions that were active in Urat1/RST KO but not wildtype mice revealed that the metabolic alterations and changes in the seemingly disparate sub-systems are actually coordinated/complementary reactions related to ROS detoxification and maintaining the redox state of the cell via peroxidation reactions relying on Vitamin C metabolism and maintenance of cofactors in the reduced state.

In order to provide an objective assessment of these alterations, a metabolite connectivity analysis of the reactions unique to KO and WT models, respectively was performed. Redox cofactor pairs (e.g., NADH/NAD, Q10H2/Q10, FADH2/FAD, NADPH/NADP) dominated the list of reactions that were present in KO but not WT (Supplementary Materials Table S2). Focusing on transported (exchange) metabolites for WT versus Urat1KO (Figure 8), there are metabolites unique to the WT as well as to the KO, with wide ranging differences in molecular size and molecular weight between them all. It would be very difficult to attribute these alterations simply to the ability to transport all of these different metabolites; however, in the context of the disruption of the redox state due to loss of the ability to transport urate, the systemic changes and shifts in metabolism to balance the redox state become clear. In order to maintain consistency between the chemoinformatics-ML and GEM-COBRA analyses, we relaxed the significance cutoff criteria of the metabolomic data to *p* < 0.1 to determine whether we still observed a dominant effect on the redox state. The pattern persisted with redox cofactors dominating the connectivity analysis (Supplementary Materials Tables S3–S6). Although from the list of altered metabolites and conventional pathway analysis it appears that many different metabolites involving many different subsystems are altered with Urat1/Rst KO (Supplementary Materials Tables), it is important to appreciate that the tie that binds these alterations is not related to a particular pathway, but rather specific metabolic processes: (1) ROS detoxification and (2) redox state maintenance (Figure 9 and Table 1). Comparison of the WT and Urat1/Rst KO models showed common themes in pathways. The analysis revealed features that included: superoxide reduction to hydrogen peroxide and maintaining the reduced states of FAD, NAD, and Q10 (Figure 9 and Table 2). Crucially, evaluation of the specific reactions in different metabolic pathways can be seen to largely involve balancing redox stresses and/or maintaining the redox state.

## 4. Discussion

High uric acid levels are associated with gout, urate kidney stones, progression of renal disease, cardiovascular disease, hypertension, and metabolic syndrome. URAT1 has generally been held to be a highly selective urate transporter [2,10,11,45]. This view is supported by in vitro transport data, human disease mutations, and GWAS studies. It is considered a major drug target for the treatment of hyperuricemia [46,47].

URAT1 (SLC22A12), originally Rst in mice, is a member of the SLC22 transporter family [48]. The SLC22 family was identified in 1997 when homologies between OAT1 (originally NKT, SLC22A6) and two other family members, SLC22A1 (OCT1) and SLC22A7 (OAT2, NLT) were discovered [49]. The family now consists of 30 or so transporters in human and/or mouse. Recently, a new evolutionarily-based sub-classification of SLC22 has placed family members into 6-8 groups which, nonetheless, seem to correlate with the more superficial, original grouping into OATs, OCTs and OCTNs [50]. Along with the closely-related OAT1 and OAT3, URAT1 falls into the OAT subclade. Nevertheless, phylogenetic analyses place it in a different subgroup (OATS3) from OAT1 and OAT3, which are in the OATS1. This leads to the notion that, while all three are important urate transporters, there may be different overall subgroup-specific metabolic functions. That view is supported by the results presented here.

While original studies of the Urat1/Rst KO mouse in 2008 also supported a role in urate transport, the alterations in urate levels were not as great as expected from certain human studies, and it was suggested at the time that Urat1 might have roles beyond the transport of urate [6]. Indeed, the untargeted metabolomics analyses in the original knockout studies raised the possibility that Urat1 might transport other endogenous metabolites, but none were clearly identified.

We have identified approximately 20 (at the less stringent cutoff of *p* < 0.1 and 8 at the cutoff of *p* < 0.05) such endogenous metabolites in the Urat1KO mice and, interestingly, there is only very modest overlap with the Oat1 metabolomics data and very minimal overlap with the Oat3 metabolomics data. In previously published work, Oat1 and Oat3 KO mice had much larger numbers of metabolites altered in comparison to WT animals; reflecting the broader spectrum of metabolic involvement, including multi-organ interactions [20,44,51]. These differences in KO metabolites are very interesting because of the fact that Urat1 is closely-related by sequence to Oat1 and Oat3, and it was the basis of our deeper exploration of Urat1 function. Our chemoinformatics and machine learning analyses demonstrated that the molecular properties of the metabolites accumulating in the Urat1KO are distinct from those accumulating in the two Oat KOs (Figure 2 and Figure 3).

However, the affected pathways based on standard pathway analysis, were not connected in an obvious way to each other or to uric acid metabolism. That led us to perform an integrated transcriptomic and metabolomic data analysis using GEMs in order to reconcile the disparate metabolic alterations into a broader portrait of changes in metabolism at the level of the kidney and the whole animal. There are have been a growing number of examples for the use of GEMs to integrate and analyze complex physiological responses to drug treatments and gene knockouts [52,53,54,55,56]. The WT and Rst KO GEMs made it possible to discover that the altered individual pathways–including those involved in metabolism of pyrimidines, sugars, fatty acids, amino acids–were related to cofactor charging and ROS species detoxification. Thus, Urat1 functions not only in uric acid homeostasis but is coupled to maintenance of the redox state.

Our analyses provide much needed physiological context to the widely-held view of uric acid as a modulator of redox state [45,46]. However, we do not propose a direct link between uric acid transport by Urat1 and our analysis of tissue and whole animal metabolic state. Instead, through the new identification of the metabolites in the Urat1KO and the corresponding GEM analyses, we were able to arrive at this more global picture of kidney and whole animal alterations in redox metabolism resulting from the loss of Urat1 function. Indeed, this is perhaps the first analysis that is able to strongly make that case.

There are other implications as well. The link to sugar and fatty acid pathways related to ROS detoxification in turn may also provide a potential mechanism for the dysregulation seen in certain types of metabolic syndrome, a common and major feature of which is altered uric acid levels. In this regard, it is noteworthy that another important human urate transporter, SLC2A9, may, as seems to be the case with URAT1, play a role in carbohydrate metabolism.

## 5. Conclusions

In summary, while URAT1 does not broadly regulate metabolites in vivo as is the case for the closely related multispecific transporters OAT1 and OAT3, neither does it appear to regulated only one metabolite, as is generally emphasized in the literature. The ability to interact with multiple substrates is apparent from the growing list of drugs and metabolites interacting with the transporter in vitro [34,57,58,59]. However, the main results of this study, involving data-driven transcriptomic and metabolomic simulation and analyses, are much more general: the metabolic alterations in the seemingly disparate pathways and sub-systems are complementary reactions related to ROS detoxification and maintaining the redox state of the cell via peroxidation reactions relying on Vitamin C metabolism as well as the maintenance of cofactors in the reduced state.

The results set the stage for a whole new range of future studies on the physiological roles of URAT1-including the potential metabolic side effects of drugs blocking URAT1 function as well as the connection between URAT1 function and pathways involved in metabolic syndrome and pathophysiological states in which the cellular redox state plays a critical role.

## Figures and Tables

**Table 1:** Selected reactions and corresponding subsystems that were present in Urat1/Rst KO models but not WT. Superoxide dismutase (SPODM) isoforms in multiple compartments, in addition to ascorbate, metabolize superoxide to hydrogen peroxide. Glutathione and NADPH provide reducing equivalents to ‘recharge’ ascorbate. Sub-script letters specify the compartment (c: cytosol, n: nuclear, m: mitochondria, e: extracellular, x: peroxisome). Abbreviations: h: hydrogen ion, o2: oxygen, h2o2: hydrogen peroxide, o2s: superoxide, ascb: ascorbate (Vitamin C), dhadascb: dihydroascorbate, gthrd: reduced glutathione).

Reaction Abbreviation	Subsystem	Biochemical Reaction	Compartment
SPODM	ROS detoxification	2 h_c_ + 2 o2s_c_ → o2_c_ + h2o2_c_	Cytosol
SPODMe	ROS detoxification	2 h_e_ + 2 o2s_e_ → h2o2_e_ + o2_e_	Extracellular
SPODMm	ROS detoxification	2 h_m_ + 2 o2s_m_ → o2_m_ + h2o2_m_	Mitochondria
SPODMn	ROS detoxification	2 h_n_ + 2 o2s_n_ → h2o2_n_ + o2_n_	Nucleus
SPODMx	ROS detoxification	2 h_x_ + 2 o2s_x_ → o2_x_ + h2o2_x_	Peroxisome
ASCBOX_1_	Vitamin C metabolism	3 h_c_ + ascb-L_c_ + 2 o2s_c_ → 2 h2o2_c_ + dhdascb_c_	Cytosol
DASCBR_1_	Vitamin C metabolism	nadph_c_ + dhdascb_c_ → nadp_c_ + ascb-L_c_	Cytosol
DHAOX_c_	Vitamin C metabolism	dhdascb_c_ + 2 gthrd_c_ → h_c_ + ascb-L_c_ + gthox_c_	Cytosol

**Table 2:** Selected reactions and corresponding subsystems with increased flux in Urat1/Rst KO relative to the WT model. Reactions that were at least 1.5 times the mean flux in KO compared to WT were selected and further filtered by removing exchange/transport reactions, exogenous metabolism, and fatty acid oxidation/synthesis. Redox cofactor balances and charges are noted in multiple reactions. Interestingly, generation of lactate as a by-product in order to produce (reduced) glutathione (GLYOX) provides a link to how the increased fluxes related to the unique Urat1KO reactions (Table 1). Sub-script letters specify the compartment (c: cytosol, n: nuclear, r: endoplasmic reticulum). Abbreviations: h2o: water, ac: acetate, cit: citrate, oaa: oxaloacetate, lac: lactate, gthrd: reduced glutathione, nad: nicotinamide adenine dinucleotide (oxidized), nadh: nicotinamide adenine dinucleotide (reduced), cmpacna: CMP-N-acetylneuraminate, crm-hs: ceramide, dag-hs: diacylglycerol, dhcrm-hs: dihydroceramide, chol: choline, pchol-hs: phosphatidylcholine, atp: adenine trinucleotide phosphate, adp: adenine dinucleotide phosphate, 4hpro-LT: 4 hydroxy-L-proline, 1p3h5c: L 1-Pyrroline-3-hydroxy-5-carboxylate, gd1a-hs: GD1a-ganglioside, gt1a-hs: GT1-ganglioside, g3pc: Glycero-3-phosphocholine, sphmyln-hs: sphingomyelin, pail: phosphatidylinositol, mi1p-D: myo-inositol 1-phosphate, lgt-S: S-lactoylglutathione).

Reaction Abbreviation	Subsystem	Biochemical Reaction	Compartment
HMR_4782	Arginine and proline metabolism	o2_c_ + h_c_ + 4hpro-LT_c_ → 2 h2o_c_ + 1p3h5c_c_	Cytosol
CITL	Citric acid cycle	cit_c_ → ac_c_ + oaa_c_	Cytosol
GPDDA1	Glycerophospholipid metabolism	h2o_c_ + g3pc_c_ → h_c_ + chol_c_ + glyc3p_c_	Cytosol
HMR_0853	Glycosphingolipid metabolism	cmpacna_c_ + gd1a-hs_c_ → h_c_ + cmp_c_ + gt1a-hs_c_	Cytosol
RE2675C2	Glycosphingolipid metabolism	o2_c_ + h_c_ + nadph_c_ + dhcrm-hs_c_ → 2 h2o_c_ + nadp_c_ + crm-hs_c_	Cytosol
PI3P5K	Inositol phosphate metabolism	atp_c_ + pail3p-hs_c_ → h_c_ + adp_c_+ pail35p-hs_c_	Cytosol
PIK3n	Inositol phosphate metabolism	atp_n_ + pail-hs_n_ → h_n_ + adp_n_ + pail3p-hs_n_	Nucleus
PIK4n	Inositol phosphate metabolism	atp_n_ + pail-hs_n_ → h_n_+ adp_n_ + pail4p-hs_n_	Nucleus
PIPLC	Inositol phosphate metabolism	h2o_c_ + pail-hs_c_ → h_c_ + dag-hs_c_ + mi1p-D_c_	Cytosol
GLYOX	Pyruvate metabolism	h2o_c_ + lgt-S_c_ → h_c_ + gthrd_c_ + lac-D_c_	Cytosol
SMS	Sphingolipid metabolism	pchol-hs_c_ + crm-hs_c_ → dag-hs_c_+ sphmyln-hs_c_	Cytosol
RE3050R	Vitamin A metabolism	h2o_r_ + nad_r_ + retinal-cis-13_r_ → 2 h_r_ + nadh_r_ + retn_r_	Endoplasmic reticulum

## Data Availability

All data described in this manuscript is available in the main text, Supplemental Material, and cited references.

## Supplementary Materials

The following are available online at https://www.mdpi.com/article/10.3390/antiox12030780/s1, Table S1: Metabolomic data, Table S2: Sampled reaction fluxes, Table S3–S6: Metabolite connectivity (WT and KO, GIMME), Table S7: Metabolite connectivity (CORDA), JSON: WT and KO models.

## Abbreviations

ANOVA	Two way analysis of variance
COBRA	Constraint-based Reconstruction Analysis
CKD	Chronic Kidney Disease
FBA	Flux Balance Analysis
FVA	Flux Variability Analysis
GEM	Genome-scale model
GIMME	Gene Inactivity Moderated by Metabolism and Expression
IACUC	Institutional Animal Care and Use Committee
KO	knockout
OAT1	Organic Anion Transporter 1
OAT3	Organic Anion Transporter 3
ROS	reactive oxygen species
RSST	Remote Sensing and Signaling Theory
WT	wildtype
URAT1	Urate transporter 1 (known as RST in mice)
